# The feasibility of a randomized controlled crossover trial to assess the effect of probiotic and prebiotic supplementation on the health of elite wheelchair athletes

**DOI:** 10.1186/s40814-023-01339-6

**Published:** 2023-06-15

**Authors:** Anneke Hertig-Godeschalk, Marija Glisic, Belinda Ruettimann, Ezra Valido, Simona Capossela, Jivko Stoyanov, Joelle L. Flueck

**Affiliations:** 1 Institute of Sports Medicine, Swiss Paraplegic Centre Nottwil, Nottwil, Switzerland; 2 Swiss Paraplegic Research, Nottwil, Switzerland; 3 Institute of Social and Preventive Medicine, University of Bern, Bern, Switzerland

**Keywords:** Bowel, Feasibility, Gastrointestinal problems, Microbiome, Paralympic, Spinal cord injury, Prebiotic, Probiotic

## Abstract

**Background:**

Gastrointestinal (GI) problems represent a health burden in Para athletes and can ultimately reduce athletic performance. This study aimed to evaluate the feasibility of a randomized controlled crossover trial (RCCT) assessing the effects of probiotic and prebiotic supplementation on the health of Swiss elite wheelchair athletes.

**Methods:**

The RCCT was conducted between March 2021 and October 2021. Athletes were randomized to receive either a daily probiotic (3 g of probiotic preparation, including eight bacterial strains), or a daily prebiotic (5 g of oat bran) supplementation first. After the first supplementation phase (4 weeks), a washout period (4 weeks) and the second crossover supplementation phase (4 weeks) followed. Data were collected at four study visits (every 4 weeks) and included 3-day training and nutrition diaries, the Gastrointestinal Quality of Life Index (GIQLI) questionnaire, stool samples, and fasting blood samples. The study assessed the feasibility criteria such as recruitment rate, retention rate, success of data collection, adherence to the protocol, willingness to participate, and safety.

**Results:**

This pilot study met the majority of the predefined minimum requirements for the feasibility criteria. Out of 43 invited elite wheelchair athletes, 14 (33%) consented (mean (standard deviation) age: 34 (9) years, eight females, 11 with a spinal cord injury). The desired sample size was not reached, but the achieved recruitment rate was modest, especially considering the population studied. All participating athletes completed the study. With the exception of one missing stool sample and two missing diaries, data were successfully collected for all athletes at all four visits. Most athletes adhered to the daily intake protocol for at least 80% of the days, both for probiotics (*n* = 12, 86%) and prebiotics (*n* = 11, 79%). Ten (71%) athletes would be willing to participate in a similar study again. No serious adverse events occurred.

**Conclusion:**

Despite the limited number of elite wheelchair athletes in Switzerland and the modest recruitment rate, the implementation of a RCCT in elite wheelchair athletes is feasible. The data collected in this study provide essential information for the design of the subsequent study which will include a larger cohort of physically active wheelchair users.

**Trial registration:**

Swiss Ethics Committee for Northwest/Central Switzerland (EKNZ), 2020–02337). ClinicalTrials.gov, NCT04659408.

**Supplementary Information:**

The online version contains supplementary material available at 10.1186/s40814-023-01339-6.

## Key messages regarding the feasibility

- The main uncertainties were the recruitment rate and the completeness of data collection.
- The key finding was that the implementation of a randomized controlled crossover trial in elite wheelchair athletes is feasible, as an adequate recruitment rate and satisfactory data collection could be achieved.
- The following implications will be considered in the design of the main study: recruiting athletes during their off- or pre-season, allowing sufficient flexibility in the scheduling of study visits, and enlarging the pool of athletes by also recruiting at a recreational level.

## Background

Gastrointestinal (GI) problems are highly prevalent and represent a health burden in Para athletes [1, 2]. Typical GI problems include bloating, nausea, and related reduced nutritional intake, which can interfere with optimal performance in able-bodied athletes and even more so in Para athletes [3, 4]. Prolonged strenuous exercise, especially in humid and hot conditions, can further increase the occurrence of GI discomfort [5, 6]. GI motility disorders, including delayed gastric emptying, and neurogenic bowel dysfunction, including constipation, are especially common in individuals with a spinal cord injury (SCI) [7, 8]. Reduced energy expenditure and energy availability, nutrient deficiencies, and prolonged intestinal transit times could also play a role in the development of GI complaints in athletes with a SCI [3, 9].

The microbiome is receiving increasing attention for its potential impact on both general health and athletic performance [10, 11]. It encompasses the microorganisms and their unique ecological niches within a given environment [11]. Notably, the microbiome is dynamic and can adapt to both dietary intake and physical activity [10, 12]. Highly trained athletes have been found to have a higher alpha diversity, indicating a greater microbiome diversity, compared to non-athletes [13]. While moderate exercise can increase the abundance of some beneficial bacteria, intense exercise can actually decrease their abundance [13]. Supplementation with living microorganisms, known as probiotics, can alter the population and structure of the gut microbiota which may ultimately improve GI health [14]. Furthermore, probiotics may alleviate stress-induced symptoms, leading to a reduction in GI distress [4]. Additionally, probiotics show promise in restoring dysbiosis in the microbiome as well as modulating the inflammatory response after a SCI [15, 16]. The exact benefits of probiotics depend on the strain, dosage, and intake method [14]. Even though effects on physiological parameters were not always found, able-bodied athletes reported clinically relevant effects of probiotic supplementation including reduced severity and duration of GI symptoms [5, 6, 14]. Ultimately, the reduction of GI symptoms with probiotic supplementation may lead to maintained or even improved athletic performance [17]. The intake of prebiotics, fermented food ingredients that can induce the activity of certain microorganisms, has also shown potential for modifying the microbiome [18]. Improved GI and immune system function but also improved mental health have been associated with prebiotics [4, 18]. Therefore, the intake of both probiotics and prebiotics may benefit athletes [4, 18]. To our knowledge, no studies have been conducted on the effects of these supplements in wheelchair athletes. Implementing intervention studies in elite athletes can be challenging due to the tight training and competition schedules. Furthermore, athletes may be reluctant to try something new due to potential adverse effects on performance, which can further complicate the implementation of such a study.

This pilot study aimed to assess the feasibility of a randomized controlled crossover trial (RCCT) of probiotic and prebiotic supplementation in Swiss elite wheelchair athletes. The feasibility criteria, including the recruitment rate, completeness of the data collection, and adherence to the intervention, were analyzed. Furthermore, we descriptively analyzed subjective GI health during the intervention period. The protocol of this study was recently published [19].

## Materials and methods

The detailed protocol of this trial has been previously published [19], and a brief summary is provided below.

### Study design, population, and sample size

This RCCT was planned following the CONSORT guidelines [20] (Additional file 1). The study was conducted at the Institute for Sports Medicine within the Swiss Paraplegic Center, which specializes in examining wheelchair athletes. Athletes aged 18 or older who have been shortlisted for the Tokyo 2020 Olympic Games or are otherwise active in (inter)national competitions were invited to participate. First, an email including the study information was sent. Next, we aimed to contact all athletes in person at the study center or by phone. Individuals diagnosed with GI diseases, being pregnant, or currently taking concomitant medication, including antibiotics, right at the start of the study were not eligible. All athletes provided informed consent as documented by a signature and were offered a financial compensation of 50 Swiss Francs (52 US dollars) per study visit. The study was conducted according to the Declaration of Helsinki and Swiss law, approved by the Swiss Ethics Committee for Northwest/Central Switzerland (EKNZ, project ID: 2020–02337), and was registered at ClinicalTrials.gov (NCT04659408).

Given the limited number of active wheelchair athletes in Switzerland, we calculated our required sample size based on recommendations for a minimized pilot trial sample size [21]. We expected to observe a small effect size in the current pilot study. With 80% power and a two-sided 5% significance, we aimed to recruit 20 athletes [21]. Further details for the sample size calculation are provided in our protocol paper [19].

### Intervention and randomization

The intervention supplement consisted of a 4-week intake of the commercially available freeze-dried multispecies probiotic preparation *Bactosan pro FOS* (Mepha, Basel, Switzerland). Athletes were instructed to take the supplement following the product information, taking one sachet (3 g) daily preferably before breakfast or at least 3 h after a meal. The control supplement consisted of a 4-week daily intake of 5 g (1 teaspoon) oat bran (Naturaplan, Coop, Switzerland), preferably with breakfast. Due to the obvious differences between both supplementations, blinding was not possible. Once per week, athletes were reminded to take the supplement. Adherence to the intervention was assessed in two ways during the visits. First, athletes were asked to return the empty packages of the supplements. Second, athletes were asked whether they took the daily supplement; if not, how many days were missed plus the reasons for not taking the supplement. Potential side effects were also assessed during the visits. Athletes were asked to keep their usual nutrition and training routine during the entire study.

Athletes were allocated to receive either the intervention (probiotic) or control (prebiotic) supplement first utilizing a 1:1 randomization with blocks of two and four, executed by a Good Clinical Practice compliant data management system (secuTrial®, interActive Systems, Berlin).

### Assessments

At the study start (T0), the following parameters were collected: age, sex, length, waist circumference, medical diagnosis and/or SCI characteristics, presence of diseases, type of sport, average number, and duration of training. Body composition was determined through a dual X-ray Absorptiometry (DXA) scan.

Study visits were planned every 4 weeks (T0–T3, ± 7 days) during the entire 12-week study duration and contained several assessments. Blood pressure and body weight were measured. The intake of medication and dietary supplements, as well as the occurrence of diseases including urinary tract infections, was assessed. The frequency of 36 GI symptoms during the previous 2 weeks was measured on a 4-point Likert scale (ranging from 0 = “all the time” to 4 = “never”) using the Gastrointestinal Quality of Life Index (GIQLI) questionnaire [22]. Leisure time physical activity intensity and duration during the last 7 days before the visit were assessed using the Leisure Time Physical Activity Questionnaire for People with Spinal Cord Injury (LTPAQ-SCI) [23]. As a proxy for strength, three maximum handgrip measurements of each hand were performed using the Jamar dynamometer following standardized procedures (Jamar Hydraulic Hand Dynamometer, Jamar, Bolingbrook, USA). Fasting blood samples were taken to analyze the following parameters: hemoglobin, ferritin, vitamin D, cholesterol, and several inflammatory and metabolic biomarkers. A stool sample was taken by the athletes within 3 days before the visit using a commercially available kit (OMNIgene®•GUT, DNA Genotek, Ottawa, Canada) and used to analyze the microbiome. Athletes were asked to fill out a detailed food and training diary for the 3 days before the visit, to assess energy intake and exercise energy expenditure. Every week during the entire study, athletes filled out the German version of the Oslo Sports Trauma Research Center (OSTRC) questionnaire [24], evaluating illness and injury as well as loss of training days during the last week. During the intervention period, athletes were reminded by email to take the daily supplement.

### Feasibility criteria

The feasibility of the pilot study was evaluated by several criteria (Table 1) based on Thabane et al. [25]: appraisal of the eligibility criteria, recruitment, consent, and retention rate; completeness of the data collection; adherence to the intervention; acceptability of the study by the athletes; resources needed for study procedures; and safety analyses, including the occurrence of serious adverse events.

**Table 1:** Overview of the feasibility and minimum progression criteria and outcomes reached during the pilot trial

	**Assessment**	**Minimum achievement for progression to a subsequent trial**	**Achieved in the pilot trial**	
**Recruitment and eligibility**	Invited athletes	N/A	43	
	Reasons for non-participation	Descriptive summary	“unknown (no response)” (*n* = 11), “no time” (*n* = 9), “not interested” (*n* = 7), “distance to study center too far” (*n* = 2)	
	Athletes willing to participate, fulfilling inclusion criteria and providing informed consent	> 25% agreed to participate	33% (*n* = 14)	**✓**
	Ineligible athletes	Descriptive summary	0	
**Retention**	The number of randomized athletes retained/who managed to complete the study protocol	> 80% of athletes at the study start	100%	**✓**
**Data collection**	Completed questionnaires	> 75% completed, collected, or analyzed at each visit	100%	**✓**
	Completed diaries		96%	**✓**
	Stool samples collected		98%	**✓**
	Successfully analyzed microbiome composition		100%	**✓**
	Fasting blood samples collected		100%	**✓**
	Successfully analyzed blood samples		100%	**✓**
	Reasons for missing data	Descriptive summary	One stool sample and two training diaries missing due to competition and related stress	
**Adherence to intervention**	Adherence to daily intake for both supplements	> 80% of days	86% for probiotics and 79% for prebiotics	**✓/✖**
**Acceptability**	Rating (from 0–10) over six items ^a^	Having an average of at least five points for each item	8.1	**✓**
	Overall study rating (0–10)	Having an average of at least five points	8.0	**✓**
	Willingness to participate again	> 50% of athletes willing to participate again	71%	**✓**
	Open feedback	Descriptive summary	− : participation costs too much time, inconvenient to visit the study center so often ** + **: interesting subject, participation is beneficial and does not cost much time	
**Resources needed**	Time needed for athletes to collect data before each visit (stool collection, diaries, questionnaires)	< 60 min for each visit	50 min	**✓**
	Time needed to perform all assessments during each visit	< 60 min for each visit	25 min	**✓**
**Safety analyses**	Number of serious adverse events	No serious adverse events related to the study intervention or other procedures	No serious events occurred	**✓**

**✓** = reached

**✖** = not reached

^a^ Items: general interest in the study subject, communication of study information and procedures, time required, intake of supplements, assessment, and contact with the study team

At the study’s end, athletes provided feedback on the acceptability of the study by filling out a brief questionnaire. The following six items were scored on a scale ranging from 0 to 10: general interest in the study subject, communication of study information and procedures, time required, intake of supplements, assessments, and contact with the study team. Athletes were also asked whether they would participate again in a similar study (yes or no) and to provide a general study rating (0–10). Open feedback could be noted down as well. Serious adverse events were defined as any untoward medical occurrence that was life-threatening or resulted in death, required hospitalization, or resulted in persistent or significant disability.

### Data preparation

The neurological level of injury, defined as the highest motor level, was categorized into tetraplegia (C1–C8) or paraplegia (T1 or lower) [26]. The American Spinal Injury Association Impairment Scale (AIS) was used to categorize the level of impairment into complete (A or B) or incomplete (C or D) [26]. Data were reorganized and analyzed by intervention phase: pre-probiotics, post-probiotics, pre-prebiotics, and post-prebiotics.

### Data analyses

Data was described descriptively by reporting the mean, standard deviation (SD), and range. Analyses were performed using Stata (StataCorp. 2017, Stata Statistical Software: release 16.0. College Station, TX: StataCorp LLC). Adherence to daily supplement intake was assessed by calculating the proportion of athletes missing a maximum of 5 days of the total 28-day intake (adherence of 23/28 days = 82%).

## Results

In this pilot study, predefined minimum achievements were accomplished for most feasibility criteria of a RCCT of probiotic and prebiotic supplementation in Swiss elite wheelchair athletes (Table 1). Further details for each criterium will be discussed in the following paragraphs.

### Recruitment, eligibility, and retention

**Fig. 1:**
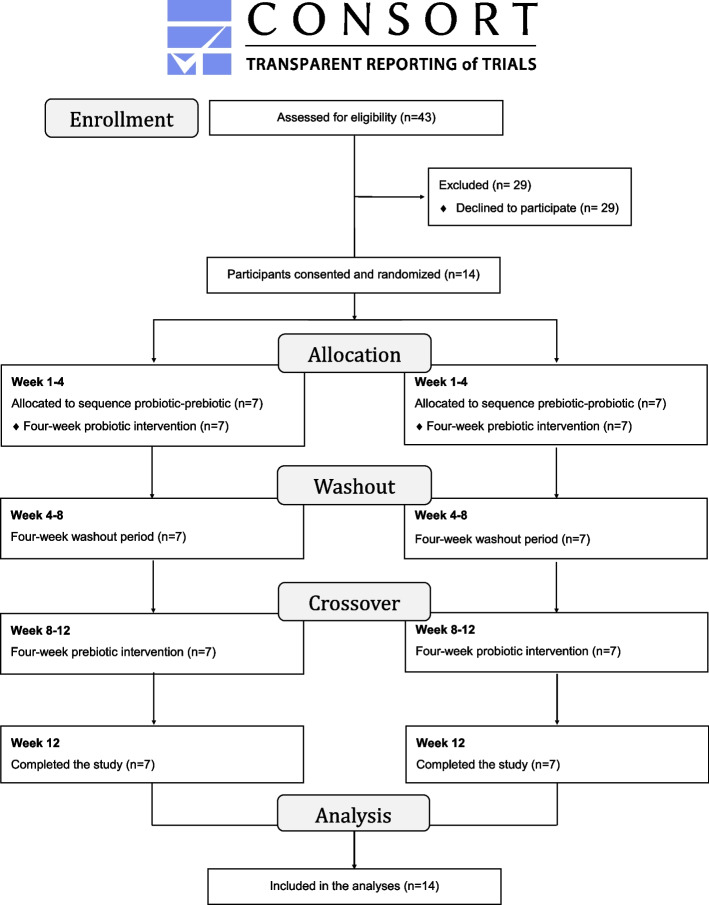
Study flow

The study flow diagram, based on Dwan et al. [27], can be found in Fig. 1. A total of 43 elite Swiss wheelchair athletes were invited to participate. Among athletes who declined participation (*n* = 29 (67%)), “no time” (*n* = 9), “not interested” (*n* = 7), and “distance to study center too far” (*n* = 2) were provided as reasons while 11 athletes did not respond to the invitation at all. Among the non-participating athletes, seven were female (24%) and all were active in summer sports. A total of 14 (33%) athletes agreed to participate (mean (SD) age: 34 [9] years, eight (57%) females, Table 2), were eligible, and provided informed consent. Most of the athletes were active in typical summer sports, which means that the study was mainly conducted during the pre-competition and competition phases. There were no dropouts which lead to a retention rate of 100%. Recruitment started in February 2021, and the last data collection was in October 2021.

**Table 2:** Athlete characteristics

	**Overall**	**Sex**	
		**Female**	**Male**
**Number of athletes**^**1**^			
*N*	14 (100)	8 (57)	6 (43)
**Age**^**2**^			
*Years*	34 (9)	32 (11)	36 (8)
**BMI**^**2**^			
*Overall* (*kg/m*^2^)	22 (4)	24 (5)	20 (2)
**SCI**^**1**^			
*Yes*	11 (79)	5 (63)	6 (100)
*No*	3 (21)	3 (38)	0 (0)
**SCI etiology**^**1**^			
*Traumatic*	5 (46)	1 (20)	4 (67)
*Non-traumatic*	6 (55)	4 (80)	2 (33)
**SCI lesion level**^**1**^			
*Tetraplegia*	4 (36)	2 (40)	2 (33)
*Paraplegia*	7 (64)	3 (60)	4 (67)
**AIS score**^**2**^			
*A*	5 (63)	2 (67)	3 (60)
*B*	1 (13)	0 (0)	1 (20)
*C*	1 (13)	0 (0)	1 (20)
*D*	1 (13)	1 (33)	0 (0)
**Diagnosis**^**1**^			
*Meningomyelocele*	5 (63)	3 (50)	2 (100)
*Multiple sclerosis*	2 (25)	2 (33)	0 (0)
*Arthrogryposis*	1 (13)	1 (17)	0 (0)
**Sport**^**1**^			
*Cycling (handbike)*	4 (29)	1 (13)	3 (50)
*Athletics (wheelchair racing)*	3 (21)	1 (13)	2 (33)
*Tennis*	3 (21)	3 (38)	0 (0)
*Others*^3^	4 (29)	3 (38)	1 (17)
**Training per week**^**2**^			
*Weekly (h)*	8.1 (2.9)	8.3 (3.5)	8.0 (2.1)

*AIS* American Spinal Injury Association Impairment Scale, *BMI* body mass index, *SCI* spinal cord injury

^1^*n* (%)

^2^Mean (standard deviation)

^3^Badminton, basketball, shooting, and skiing

### Data collection

Except for one visit of two different athletes, training, and nutrition diaries were collected for all athletes for all visits (54/56, 96%). All questionnaires were completed by all athletes during each visit. A fasting blood sample was collected from all athletes during all four visits. Each blood sample was successfully collected, processed, and analyzed. Stool samples were successfully collected from all athletes for all four visits, except for one missing sample from one athlete due to competition (55/56, 98%). Microbial DNA and microbiome sequence data could be successfully extracted for all 55 (100%) samples.

A total of 8 out of 42 (19%) visits took place more than 7 days outside of the planned four-weekly schedule. The visits had to be rescheduled due to competitions, sickness, or other scheduling problems (range − 9 to + 80 days).

### Adherence to the intervention

A 100% adherence, meaning daily intake, was achieved in nine (64%) athletes for probiotics and six (43%) athletes for prebiotics (Table 3). The majority of athletes adhered to the daily intake for at least 80% of the days, both for probiotics (*n* = 12, 86%) and prebiotics (*n* = 11, 79%). Forgetfulness was the main reason athletes missed intake of both probiotics (*n* = 3, 60%) and prebiotics (*n* = 5, 63%).

**Table 3:** Adherence to supplement intake

	**Probiotics (*n* (%))**	**Prebiotics (*n* (%))**
**Missed supplement intake**		
*0 days*	9 (64)	6 (43)
*1–5 (4–18%) days*	3 (21)	5 (36)
*6–10 (21–36%) days*	1 (1)	2 (14)
*11–14 (39–50%) days*	1 (1)	1 (1)
**Reason for missed intake**		
*Forgot*	3 (60)	5 (63)
*Being ill*	1 (20)	0
*Intolerance/side effects from the supplement*	0	1 (13)
*Others*	1 (20)	2 (25)

Reported as the number of athletes (*n* (%))

### Acceptability of the study

The mean score over the six individual items was very satisfactory (8.1). Ten (71%) athletes would be willing to participate again in a similar study. The overall study was rated very high (8.0). As negative feedback, two athletes mentioned that participation was costing too much time, and another one wrote it was inconvenient to visit the study center so often. As positive feedback, three athletes mentioned that the study subject was interesting, and one athlete wrote that participation was beneficial while not costing too much time.

### Resources needed

Athletes needed approximately 50 min to collect data between each visit: 3 × 5 = 15 min to fill out the OSTRC questionnaire, 5 min to collect the stool sample, and 3 × 10 = 30 min to fill out the diaries. Each visit at the study center lasted around 25 min: 5 min for drawing a blood sample, 5 min to collect the stool samples and diaries from the athlete and discuss potential unclarities, and 15 min to perform all further assessments and fill out the questionnaires.

### Safety

No serious adverse events occurred during the study. Only one athlete reported minor side effects during the prebiotic supplementation, including nausea and bloating. Since this athlete suffered from a urinary tract infection and was prescribed several medications, including antibiotics, the reason for the reported side effects might also lie here.

### GI symptoms

**Fig. 2:**
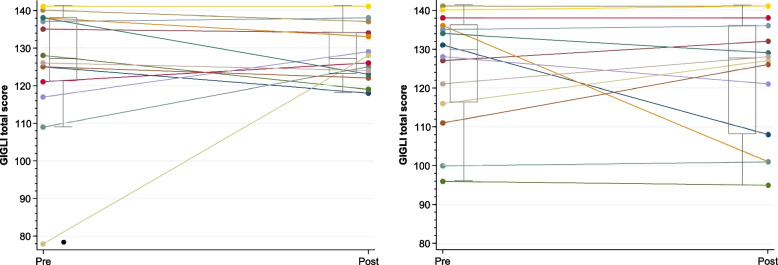
Gastrointestinal Quality of Life Index (GIQLI) scores pre and post (**A**) probiotic and (**B**) prebiotic supplementation

GI symptoms, as measured by the GIQLI questionnaire, were relatively similar pre- and post-intervention (Fig. 2). At the beginning of the study, the mean score was 122 (SD 17, range 78–141) points. In athletes with a SCI (*n* = 11), the mean score at the beginning of the study was 122 (SD 18, range 78–140) points.

## Discussion

Our study showed that the implementation of a RCCT to assess the effect of probiotic and prebiotic supplementation is feasible in elite wheelchair athletes. Except for the adherence criterium, all our predefined feasibility criteria could be achieved in this pilot trial (Table 1). Overall, high rates for data collection, processing, and sequencing were achieved.

Based on successfully fulfilling our progression criteria regarding recruitment and eligibility, there seems to be no need to reconsider the eligibility criteria. Likewise, with regard to most other criteria, there seems to be no need to modify our protocol. The only criterion that was not entirely achieved was adherence to the prebiotic supplement intake. Nevertheless, our criterion of 80% was almost reached: 79% of athletes missed a maximum of 5 days of daily intake. For the probiotic intake, 86% missed a maximum of 5 days of the 28-day intake. Forgetfulness appeared to be one of the main reasons for the lower adherence. Perhaps adherence could be further improved by daily reminders (e.g., mobile application). Another strategy could be to better inform the athletes about the potential benefits and improve the subjective perception of the prebiotic intake. Yet, daily intake might remain difficult to achieve, especially for athletes who have a busy training and competition schedule. A weekly supplementation intake could improve adherence, though this could also lead to more side effects.

Visits in four-weekly intervals are difficult to plan for elite athletes. To improve this, study participation should be preferably planned during the off-season. This will also reduce the chance of potential side effects interfering with performance during competition. Furthermore, the flexibility in the scheduling of visits should be expanded, for example, to ± 14 days. As only athletes who participated in summer sports were ultimately invited, it was not possible to make any assumptions about whether conducting this study during the off-season would increase the likelihood of participation or improve adherence to the study protocol.

The mean (SD) GIQLI score at the study start in all of the athletes (122 [17] points) but also in athletes with a SCI (122 [18] points) in our study was similar to that found in healthy individuals (126 [13] points with *n* = 168 [28] and 121 points with *n* = 150 [29]). Due to the generally high prevalence of GI problems in individuals with SCI [3, 9], we did not expect the GIQLI score to be comparable to that of able-bodied individuals. Further studies should investigate whether athletes with a SCI perhaps have fewer GI complications compared to non-athletes with a SCI. Since the GIQLI questionnaire only provides an overall score, we were not able to compare specific GI symptoms with other studies. It may be that athletes with a SCI have more GI problems in specific areas, including bowel dysfunction. This specific aspect should be further investigated in future studies.

### Strengths and limitations

Despite potential pitfalls when implementing a study in elite athletes, including time constraints and the fear of trying something novel that could affect performance, we were able to achieve a modest recruitment rate with females being more likely to volunteer. Nevertheless, time and overall burden for athletes could be reduced by implementing mobile applications for collecting data. This could also reduce the time needed for data analyses since no manual data entry is required.

A limitation of this study is the restricted number of elite wheelchair athletes in Switzerland, which confines the number of athletes that could be recruited for this study. Also due to a modest recruitment rate (33%), the calculated sample size of the pilot trial (*n* = 20) [19] could not be achieved. Although we did not specify the sample size as a feasibility criterium, we recognize that the interpretation of our feasibility criteria and the generalization of our results should be done with caution due to the small sample size. To increase the pool of athletes, recruiting individuals from a recreational level should be considered.

Adherence to the supplement intake and planning of the visits could be further optimized. Only recruiting athletes during the pre-competition could improve adherence to the supplement intake and study protocol, as potential interference with competitions could be avoided.

Although individuals with GI disease were excluded from our study, we did not use the presence of GI symptoms as an inclusion criterion. To increase the potential for improvement in GI symptoms as measured by the GIQLI score, future studies may benefit from including only individuals with GI symptoms at the beginning of the study. Given that antibiotic use is relatively common in wheelchair athletes [30], we decided not to exclude athletes who started taking antibiotics during the study. While we assessed alcohol and antibiotic intake during the study, we did not specifically control for or assess foods that may affect the microbiome, such as artificial sweeteners and foods high in phytochemicals [31]. Considering our crossover study design, potential bias from this was also minimized. However, it may be prudent to control for certain food and medication intake in future studies.

Although probiotic supplementation has shown ergogenic effects in able-bodied athletes [14], besides handgrip strength, we did not include performance assessments in our study for several reasons. We wanted to include athletes from all different sports, making direct performance assessment during training or competition challenging. It is also difficult to define one physical test that could show the ergogenic effect on these different athletes. Furthermore, to increase the potential recruitment rate, we recruited among all available wheelchair elite athletes which meant recruiting athletes during different training phases, including the pre-competition and the competition phases. This makes potential performance comparisons even more complicated.

#### Conclusion and clinical implications

Our study showed that the implementation of a RCCT to assess the effect of probiotic and prebiotic supplementation is feasible in elite wheelchair athletes. Recruiting elite wheelchair athletes may be challenging, but we have still managed to achieve a modest recruitment rate. Data were successfully collected at all four study visits, and athletes expressed high satisfaction with the study. This pilot trial provided helpful information to set up a subsequent larger-scale trial. Extending the target population beyond elite athletes, including recreational athletes and other physically active wheelchair users, should allow for an ample recruitment rate.

## Supplementary Information

**Additional file 1.** CONSORT checklist.

## Data Availability

The datasets used and analyzed during the current study are available from the corresponding author upon reasonable request.

## Abbreviations

AIS: American Spinal Injury Association Impairment Scale
DXA: Dual X-ray absorptiometry
GI: Gastrointestinal
GIQLI: Gastrointestinal Quality of Life Index
LTPAQ-SCI: Leisure Time Physical Activity Questionnaire for People with Spinal Cord Injury
OSTRC: Oslo Sports Trauma Research Center
RCCT: Randomized controlled crossover trial
SCI: Spinal cord injury
SD: Standard deviation
